# Olfactory bulb SARS‐CoV‐2 infection is not paralleled by the presence of virus in other central nervous system areas

**DOI:** 10.1111/nan.12752

**Published:** 2021-08-09

**Authors:** Gianluca Lopez, Cristina Tonello, Ganna Osipova, Luca Carsana, Mara Biasin, Gioia Cappelletti, Alessandro Pellegrinelli, Eleonora Lauri, Pietro Zerbi, Roberta Simona Rossi, Manuela Nebuloni

**Affiliations:** ^1^ L. Sacco Department of Biomedical and Clinical Sciences University of Milan Milan Italy; ^2^ Pathology Unit L. Sacco Hospital Milan Italy

The ongoing SARS‐CoV‐2 pandemic has proven to be challenging, in both clinical and pathological terms. The viral tropism for angiotensin‐converting enzyme 2 (ACE‐2) receptor‐expressing cells is paralleled in the clinical setting by the development of a spectrum of manifestations, most notably acute respiratory distress syndrome (ARDS).^1^, ^2^, ^3^ Neurological symptoms are frequent in COVID‐19 patients; however, central nervous system (CNS) infection and subsequent neurological disease attributable to SARS‐CoV‐2 remains a still poorly defined topic.^4^, ^5^ Both glial and neuronal cells have demonstrated ACE‐2 expression and are therefore susceptible to SARS‐CoV‐2 infection.^6^, ^7^, ^8^ Moreover, the possible spread of the virus through olfactory nerve fibres to the CNS remains an interesting although elusive issue.^9^

A number of post‐mortem neuropathological studies have reported vascular, thrombotic, and ischaemic alterations in COVID‐19 cases.^10^, ^11^, ^12^, ^13^, ^14^, ^15^, ^16^ Of note, the presence of SARS‐CoV‐2 within CNS specimens was reported to range between 0% and 53% of analysed cases across studies, including the olfactory bulbs and/or cerebral parenchyma.^12^, ^13^, ^14^, ^15^, ^16^

Our group aimed to describe the neuropathological findings in SARS‐CoV‐2 infected patients who died during the COVID‐19 pandemic and, by using real‐time (RT) PCR and immunohistochemistry (IHC), to define and quantify the presence of virus in selected areas showing pathological signs, as well as in areas of interest that might define a route of spread for the virus in the CNS.

A total of 15 consecutive autopsies performed in patients with demonstrated or suspected SARS‐CoV‐2 infection (positive RT‐PCR analysis of throat swab or suspected clinical course without molecular confirmation) in L. Sacco Hospital, Milan, Italy, for which the brain was obtained were included in our study. Among them, 13/15 cases had positive SARS‐CoV‐2 RT‐PCR throat swab and the remaining 2/15 demonstrated viral infection by PCR analysis on post‐mortem specimens from the brain or other organs.

The entire brain was fixed in neutral‐buffered formalin for a minimum of 21 days (range 21–42 days). Macroscopic examination and sampling were performed by cutting the brain in the coronal plane at level of the mammillary bodies and then sectioning at 1 cm intervals in order to comprehensively evaluate brain parenchyma and related structures. An extensive sampling of the brain was performed, which included areas from cervical spinal cord, brain stem (medulla oblongata, pons and midbrain), cerebellar hemispheres (cortex and dentate nuclei), cerebral hemispheres (frontal, parietal, temporal, and occipital cortex; hippocampus with entorhinal cortex, basal ganglia, thalamus, hypothalamus, amygdala, olfactory tubercles with medial and lateral olfactory tracts), and olfactory bulbs. Other organs, that is, lungs, heart, liver, kidneys, spleen, and bowel, were extensively sampled for diagnostic purposes and were routinely examined.

For histopathological examination, 3 μm thick sections from each brain paraffin block were stained with haematoxylin and eosin. IHC was performed on the most representative areas in cases with signs of inflammation, with antibodies to glial fibrillary acidic protein (GFAP‐clone EP672Y), macrophages (CD68‐clone KP1), T‐lymphocytes (CD3‐clone 2GV6), B‐lymphocytes (CD20‐clone L26), and granulocytes (myeloperoxidase‐polyclonal antibody). All antibodies were ready‐to‐use (Ventana, Roche Diagnostics, Basel, Switzerland) and staining was done with the BenchMark Ultra IHC/ISH System (Roche, Basel, Switzerland) in accordance with the standard protocols supplied by the manufacturer. Additional IHC and special stains were used as appropriate.

Neuropathological parameters assessed included gliosis, microglial activation, perivascular lymphocytic cuffing, red neurons, thrombi, and haemorrhage. Gliosis was assessed semi‐quantitatively as mild, moderate, and severe based on astrocytic morphology and GFAP expression.^17^ Microglial proliferation was assessed semi‐quantitatively as mild, moderate, or severe on the basis of microglial density, CD68 expression, and microglial nodule formation.

Selected areas with vascular and inflammatory alterations were tested via RT‐PCR to assess the presence of SARS‐CoV‐2, as well as the olfactory bulbs, olfactory tubercles with medial and lateral olfactory tracts, medulla oblongata, pons, amygdalae, hippocampi with entorhinal cortices, and hypothalamus. RNA was extracted from three 5 μm paraffin sections using Quick‐RNA FFPE Miniprep (Zymo research). Two target genes, RNA‐dependent RNA polymerase (RdRP gene, Co‐Diagnostic, Salk Lake City, Utah, USA) and envelope (E gene, the WHO/Charité, Berlin, Germany), were amplified and tested in all selected samples. All olfactory bulbs and olfactory tubercle/medial olfactory tract/lateral olfactory tract samples, as well as all other areas positive for E‐gene and/or RdRp gene, underwent additional quantitative testing with one‐step reverse transcription and qPCR of two Nucleocapsid viral regions (N1 and N2), using the GoTaq® 1‐Step RT‐qPCR System (Promega, Madison, WI, USA) and the 2019‐nCoV CDC Diagnostic Panel (IDT, Coralville, IA, USA) according to manufacturer's instructions. The N1 and N2 viral regions were detected in 10‐μl volume reactions using 0.2 μg of purified RNA. Reactions were performed on a CFX96 (Bio‐Rad, CA, USA) according to the following thermal profile: initial denaturation (95°C, 10 min) followed by 45 cycles of 15 s at 95°C (denaturation) and 1 min at 60°C (annealing‐extension). A melting curve analysis was assessed for amplicon characterisation. Viral copy quantification was assessed by creating a standard curve from the quantified 2019‐nCoV N positive Plasmid Control (IDT, Iowa, USA). Cycle threshold values less than 35 were considered as positive test results. Human RNase P was used as an internal control to confirm that RNA was adequately conserved and extracted^3^; in addition, appropriate positive and negative tissue samples were used as controls. SARS‐CoV‐2 PCR for E‐gene and RdRP gene was also performed in lung, heart, kidney, and liver tissues from each case.

Areas positive with RT‐PCR testing underwent IHC for SARS‐CoV‐2 spike glycoprotein (Abcam, ab272420, Cambridge, United Kingdom),^18^ as described by the manufacturer. Lung and placental tissue from cases with proven SARS‐CoV‐2 infection were used as positive controls. Lung tissue from an autopsy of 2017 was used as negative control.

Our results are summarised in Table 1. Clinically, 3/15 patients (20%) presented with neurological symptoms of new onset: one with tetraparesis, ataxia, dysautonomia, and ophthalmoplegia; one with seizures and aphasia; one with seizures alone. Two other patients had dementia, which started long before SARS‐CoV‐2 infection.

**TABLE 1:** Clinical and neuropathological characteristics of 15 patients who died with SARS‐CoV‐2 infection

Case ID	Gender	Age at death	Covid swab positivity	Neurological symptoms	Brain weight (g)	Gross findings	Neuropathological findings	Lung pathology	Olfactory bulbs						Other CNS areas	IHC	Other organs with RT‐PCR positivity	Other organ findings
									E‐gene	RdRp gene	N1 gene	N1 gene quantification (copies/μl)	N2 gene	N2 gene quantification (copies/μl)				
1	M	56	+	0	1300	Subarachnoid and cerebellar parenchymal haemorrhage	Medulla: moderate gliosis; cortical white matter: mild gliosis and microglial activation; subarachnoid and cerebellar parenchymal haemorrhage	DAD (all phases) and diffuse microthrombi	−	−	−	−	−	−	0	np	Lung, heart	Myocarditis
2	M	70	+	0	1260	0	Cerebellum: plurifocal red neurons (Purkinje cells)	Proliferative and organising DAD	−	−	−	−	−	−	0	np	Heart, lung	Myocardiosclerosis, small vessels pulmonary thromboembolism
3	F	73	+	Tetraparesis, ataxia, dysautonomia, ophthalmoplegia	1260	0	Brainstem: mild gliosis, microglial activation; cerebellum: microglial activation; CA1: focal red neurons	Focal exudative DAD	−	−	24.74	508	24.25	1798	0	Olfactory bulbs	0	Small vessel pulmonary thromboembolism
4	M	51	+	0	1410	Petechiae in corpus callosum	Cerebellum: focal red neurons (Purkinje cells); corpus callosum: microhaemorrhages; prefrontal cortex, left: focal ferruginized neurons	Exudative and proliferative DAD; focal microthrombi	−	34.5	−	−	−	−	0	0	Lung, kidney	None
5	M	67	+	0	1390	Parenchymal cerebral haemorrhage, cingulate gyrus and parietal lobe, right	Parietal cortex and cingulate gyrus: left microhaemorrhages; basal ganglia, left: leptomeningeal thrombus; middle cerebral arteries: thrombi	Exudative and proliferative DAD; diffuse microthrombi	30.2	29.7	31.81	2	30.5	15	0	0	Lung, kidney, liver	Myocardiosclerosis, small vessels pulmonary thromboembolism
6	F	97	+	Dementia	1050	Atrophy	Midbrain: focal red neurons	Focal exudative and proliferative DAD	−	−	−	−	34.17	1	Midbrain: E‐gene: 34.9	0	Lung, liver, kidney, heart	Chronic ischaemic cardiomyopathy, myocardialamyloidosis
7	F	74	+	Seizures, aphasia	1150	0	Brainstem, cerebellum: focal red neurons; hippocampus and amygdala, left: mild gliosis and microglial activation; amygdala, left: necrosis with neutrophilic infiltrate; middle cerebral arteries: thrombi	Aspergillus pneumonia and plurifocal microthrombi	−	−	26.85	94	26.53	315		0	Lung, heart, kidney	Myocardiosclerosis, small vessels pulmonary thromboembolism
8	M	62	+	0	1420	Parenchymal plurifocal haemorrhages, cerebral and cerebellar, inundation of third ventricle	Pons, midbrain, cerebellum, cerebral hemispheres: microhaemorrhages	DAD (all phases) and diffuse microthrombi	−	−	−	−	−	−	0	np	Lung, heart, liver	Small vessel pulmonary thromboembolism, lymphocytic myocarditis
9	M	51	+	0	1220	0	0	Proliferative DAD and plurifocal microthrombi	33	32.7	−	−	30.95	11	0	0	Lung, heart, kidney, liver	Myocardiosclerosis
10	M	47	−	Seizures	1420	White matter discoloration; cerebellar petechiae	Grey and white matter, diffuse: plurifocal petechial haemorrhages, moderate gliosis, microglial activation, necrosis, neutrophilic infiltrate	Focal exudative DAD	−	−	−	−	−	−	0	np	Lung, heart, kidney, liver	None
11	F	4	+	0	1340	Plurifocal parenchymal haemorrhages, cerebral and cerebellar; marked oedema	Cerebellum: focal red neurons (Purkinje cells) and microhaemorrhages; cerebral cortex and brainstem: focal red neurons, microhaemorrhages and diffuse, marked oedema	Exudative DAD	−	−	−	−	−	−	Midbrain: E‐gene 33.5	0	Lung, intestine	Intestinal infarction, MIS‐C
12	M	62	+	0	1180	Brain stem and cerebellar haemorrhage	Brainstem, cerebellum, cerebral cortex: plurifocal red neurons; cerebellum: microhaemorrhages	DAD (all phases)	30.7	32.2	29.46	12	29.19	41	0	0	Lung	None
13	F	85	+	Dementia	880	Marked atrophy, midbrain haemorrhage	Cerebellum and midbrain: microhaemorrhages	Focal proliferative DAD	−	−	−	−	−	−	0	np	0	None
14	F	17	Not performed	0	1230	Cerebellar tonsil herniation	Prefrontal cortex, left: calcifications	Mild interstitial pneumonia oedema, haemorrhage	33.2	30.68	32.47	1	31.31	8	0	0	0	Severe acute pyelonephritis
15	M	57	+	0	1250	0	Medulla: focal perivascular lymphocytic cuffing; cerebral cortex and brainstem: mild gliosis, plurifocal microhaemorrhages	Oedema, haemorrhage	−	−	HK Neg	HK Neg	HK Neg	HK Neg	0	np	0	Acute myocardial infarction

Abbreviations: CNS, central nervous system; DAD, diffuse alveolar damage; MIS‐C, multisystem inflammatory syndrome in children; HK, housekeeping gene; IHC, immunohistochemistry; NA, not available; np, not performed.

The neuropathological spectrum in our series comprises predominantly ischaemic and/or haemorrhagic alterations and a degree of reactive change, with no evidence of viral encephalitis (i.e., no perivascular lymphocytic cuffing with concomitant microglial activation) or viral inclusions. Parenchymal haemorrhages were observed in 9 (66.6%) cases and subarachnoid haemorrhage in 2 (12.7%) cases. Signs of acute neuronal ischaemic injury (i.e., red neurons) were present in 7/15 (46.7%) cases. Areas of mild–moderate gliosis were found in 5/15 cases (33.3%). Microglial proliferation was demonstrated in 4/15 (26.7%) cases (all four had concomitant gliosis). A single case showed focal perivascular lymphocytic cuffing, without signs of gliosis or microglial proliferation. Two cases (12.7%) had thrombi in the middle cerebral arteries. One case demonstrated a thrombus in a leptomeningeal vein. Another single case demonstrated diffuse neutrophil infiltration of brain parenchyma and microhaemorrhages, with microglial proliferation and moderate gliosis. The most representative neuropathological findings are illustrated in Figure S1.

Using four different RT‐PCR assays (i.e., E, RdRp, N1, and N2 genes), the olfactory bulbs demonstrated the presence of SARS‐CoV‐2 in 8/15 (53.3%) cases: four samples (26.7%) were positive for the E‐gene, five (33.3%) for the RdRp gene, five (33.3%) for the N1 gene, and seven (46.7%) for the N2 gene. This finding was not paralleled in the next area of connection in the olfactory pathway (olfactory tubercles/lateral olfactory tract/medial olfactory tract), which tested negative in all cases. The midbrain of cases no. 6 and no. 11 tested positive for E‐gene only. All other selected samples of all cases, including olfactory‐related areas and selected areas with signs of inflammation and/or haemorrhages, tested negative using molecular assays. Using quantitative RT‐PCR, viral copies in the olfactory bulbs ranged from 1 to 508 copies/μl (mean 123.4) with N1 gene and from 1 to 1798 copies/μl (mean 312.7) with N2 gene.

Histopathological examination of the eight RT‐PCR positive olfactory bulbs was unremarkable. IHC for SARS‐CoV‐2 spike glycoprotein demonstrated a paranuclear localisation of the virus in endothelial cells of 1/8 olfactory bulb samples (Figure 1). The two midbrains showing positivity for E‐gene showed focal neuronal hypoxic‐ischaemic changes (Figure 2). IHC was negative for the virus in both midbrain samples.

**FIGURE 1:**
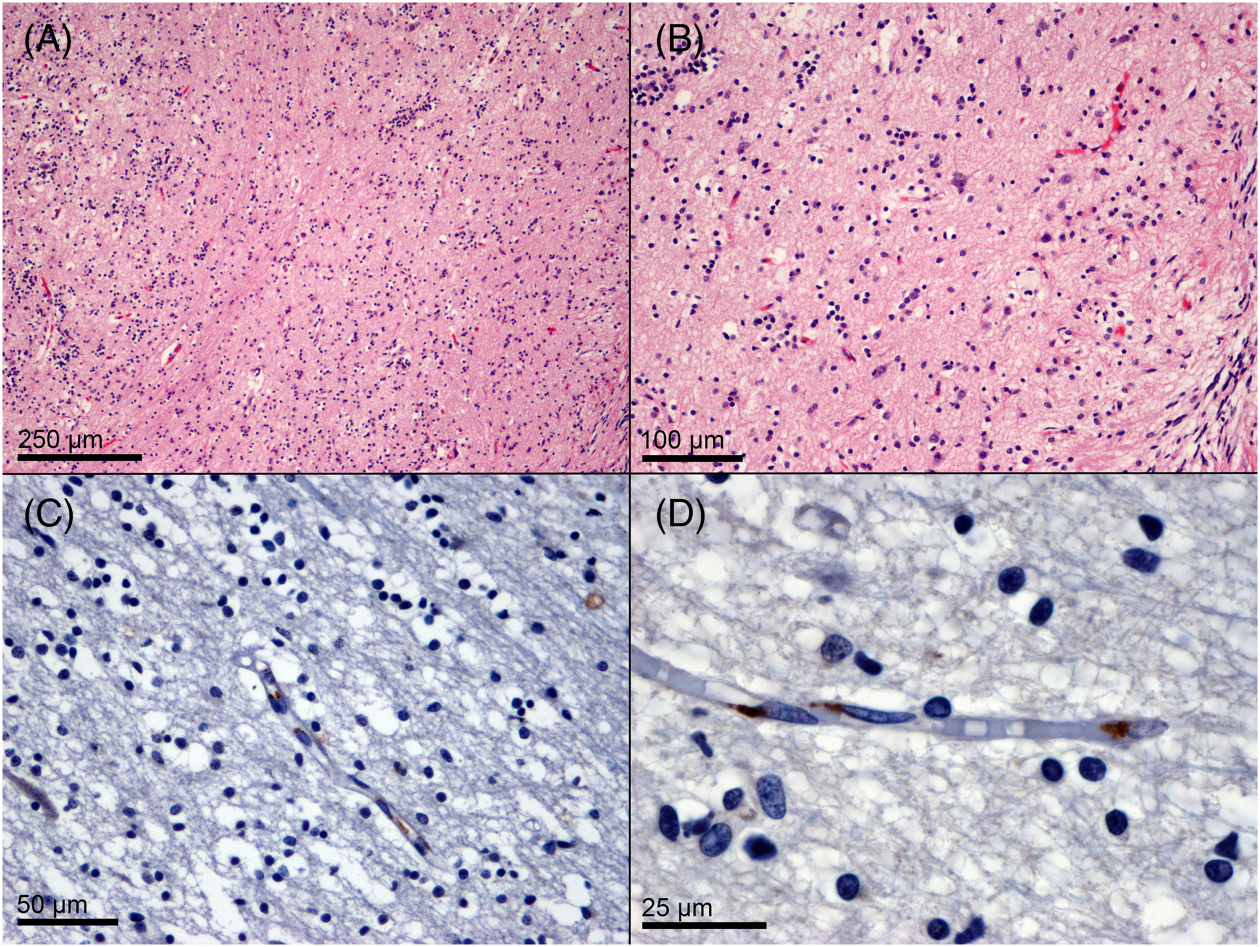
Histology and immunohistochemistry of SARS‐CoV‐2 RT‐PCR positive olfactory bulbs. Neuronal, glial, and vascular structures demonstrate no pathological features (A, H&E, 100×; B, H&E, 200×). Immunohistochemistry for SARS‐CoV‐2 spike glycoprotein demonstrates a paranuclear localisation within endothelial cells (C, anti‐SARS‐CoV‐2 spike glycoprotein, 400×; D, anti‐SARS‐CoV‐2 spike glycoprotein, 1000×)

**FIGURE 2:**
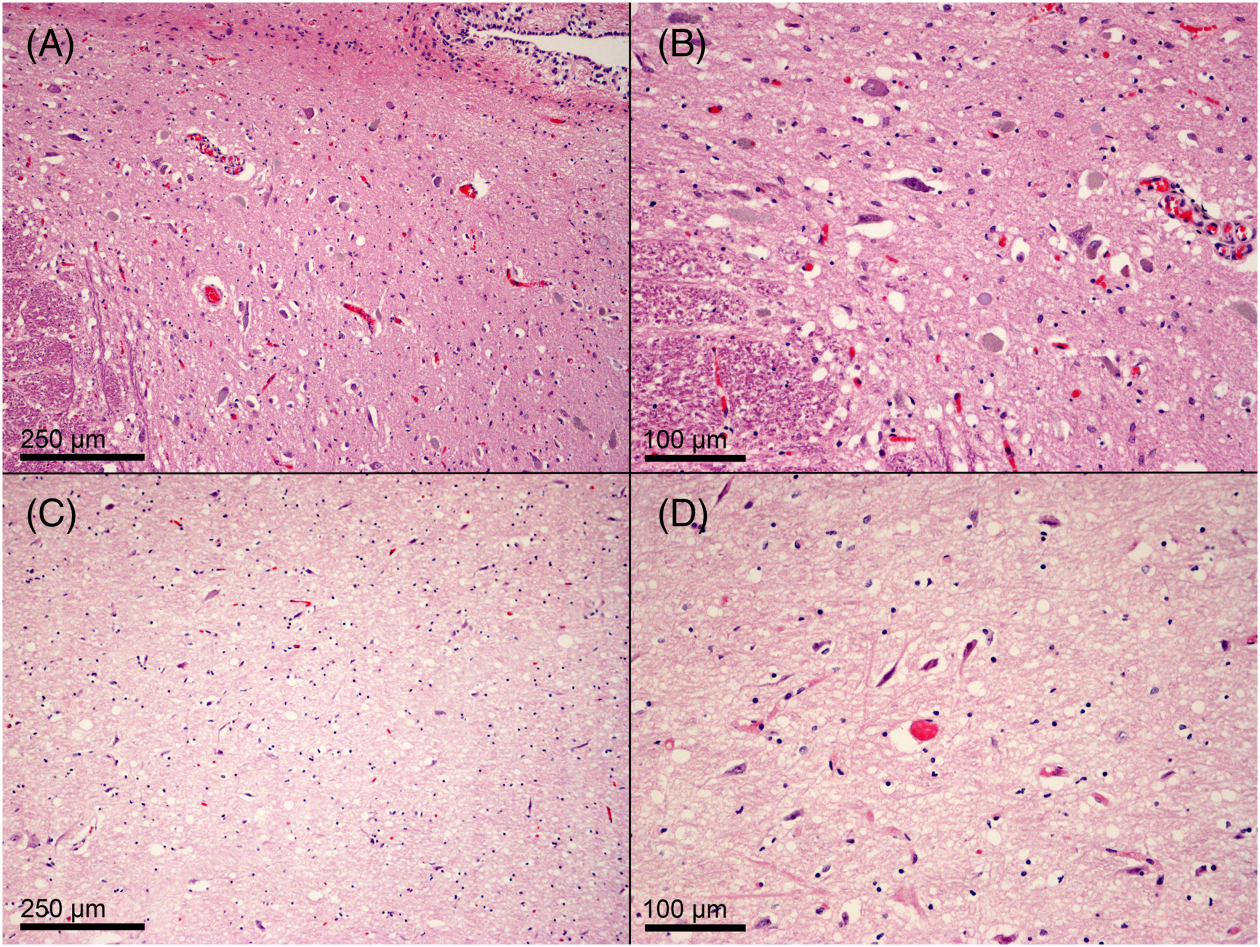
Histopathology of two midbrains with borderline positivity for SARS‐CoV‐2 E‐gene RT‐PCR. Both case no. 6 (A, H&E, 100×; B, H&E, 200×) and case no. 11 (C, H&E, 100×; D, H&E, 200×) showed focal hypoxic‐ischaemic neuronal changes, with shrinkage, hypereosinophilia, and loss of nuclear contours

Other cases showing areas of gliosis, microglial activation, and haemorrhages tested negative with RT‐PCR. Such haemorrhages and inflammatory changes could be explained by systemic haemodynamic, coagulative, or inflammatory alterations caused by SARS‐CoV‐2.^19^

Of the cases presenting with neurological symptoms, 2/3 (66.6%) showed positivity for SARS‐CoV‐2 in their olfactory bulbs, with high viral concentrations (case no. 3: N1 gene, 508 copies/μl; N2 gene, 1798 copies/μl; case no. 7: N1 gene, 94 copies/μl; N2 gene, 315 copies/μl). Among these, only one case showed positivity for SARS‐CoV‐2 IHC. Notably, all areas with gliosis, microglial activation, microhaemorrhages, and other pathological changes tested negative using RT‐PCR.

The olfactory bulb positivity in a subset of samples seems to indicate that SARS‐CoV‐2 can spread through the olfactory nerve fibres from the nasal cavity. However, the absence of virus within the neural and glial compartments in olfactory bulb samples, as well as in olfactory tubercles/lateral olfactory tract/medial olfactory tract, along with the endothelial localisation of the virus in such samples, seem to indicate that the virus spreads through a haematogenous route, which could be related to the common arterial supply of the olfactory fibres and olfactory bulb.^20^ A systemic haematogenous spread, albeit plausible, would have resulted in a wider viral spread in other CNS areas.

The midbrain of 2 (12.7%) cases showed positivity for E‐gene using RT‐PCR. Both cases showed scant neuropathological features (focal hypoxic‐ischaemic neuronal changes, Figure 2). Moreover, their ct values were relatively high (case no. 6: 34.9; case no.11: 33.5), as well as the olfactory bulb of case 4 (RdRp gene: 34.5) and case 6 (N2 gene: 34.17). Those facts, along with the negative results for the other three genes in each case, as well as for SARS‐CoV‐2 spike protein IHC, render these findings equivocal.

Unfortunately, in our cohort of patients, IHC demonstrated poor sensitivity for detecting viral particles in RT‐PCR‐positive samples, even if positive controls (infected lung and placenta) showed satisfactory results. The discrepancy may be due to the long fixation of the CNS specimens (range 21–42 days), but this explanation is not completely convincing since our positive controls also underwent long fixation periods, as well as brain specimens in other studies which used FFPE samples.^10^, ^13^

Our findings raise concerns regarding the equivalence of different genes of SARS‐CoV‐2 in PCR testing for the virus in the CNS. Further studies are needed to compare the performance of the genes E, RdRP, N1, and N2 in this setting, and clinicians and researchers should be aware that a negative result for a single gene does not preclude a positive result using another, and vice versa. The exact criteria to define the overall positivity for SARS‐CoV‐2 in this setting remains to be established.

## CONFLICT OF INTEREST

The authors declare no conflict of interests.

## ETHICS STATEMENT

The Ethics Committee of Luigi Sacco Hospital approved the use of patient data for scientific research related to the disease. This study followed the Italian general rules used for scientific research purposes (regulation no. 72–26/03/2012).

## AUTHOR CONTRIBUTIONS

GL was responsible for the original draft preparation, data collection and curation, and final draft preparation; CT, GO, EL, MB, and GC were responsible for the data collection and curation; LC, AP, and RSR performed autopsies; PZ performed autopsies and was responsible for the data collection and curation; MN performed autopsies and was responsible for the supervision, data collection and curation, and final draft preparation. All authors approved the final version of the manuscript.

## Supporting information


**Figure S1.** Gross and microscopic findings in central nervous system tissues of SARS‐CoV‐2 infected patients.
Panel A. Case n.8 demonstrated grossly intraparenchymal bilateral multifocal haemorrhages, one of which extended to the cerebral ventricles.
Panel B. Gross examination of case n. 10 revealed multiple areas of white matter discoloration (arrows).
Panel C. Histology of case n.5 demonstrated a thrombus in a leptomeningeal vein. This was the only thrombosis‐related finding in our series (H&E, 40x).
Panel D and E. Microscopically, areas of white matter discoloration of case n. 10 contained microhaemorrhages (H&E, 200x) and acute inflammatory cells parenchymal infiltrates (H&E, 400x); Panel F and G. CD68‐KP1 immunostaining demonstrated microglial proliferation (F, anti‐CD68‐KP1, 200x), and immunohistochemistry for myeloperoxidase (G, anti‐MPO, 400x) highlighted the neutrophilic infiltrate in brain parenchyma.

## Data Availability

The data that support the findings of this study are available from the corresponding author upon reasonable request.
