# Supplementary figures and images for: Olfactory bulb SARS‐CoV‐2 infection is not paralleled by the presence of virus in other central nervous system areas
Source: Neuropathol Appl Neurobiol. 2021 Aug 9;48(1):e12752. doi: 10.1111/nan.12752 (PMC8447476; doi:10.1111/nan.12752)

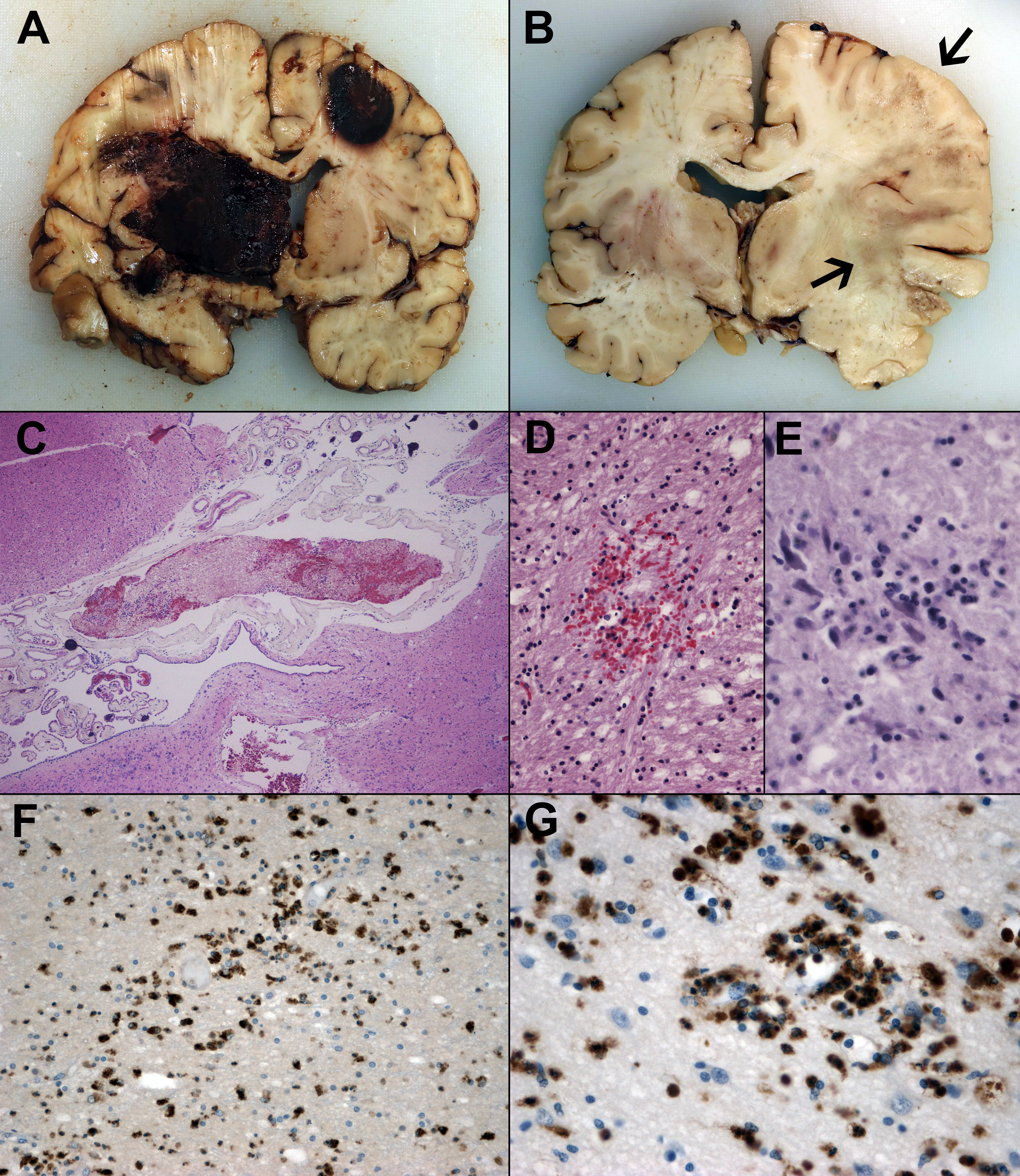

Supplement: Supplementary file 1 — Figure S1. Gross and microscopic findings in central nervous system tissues of SARS‐CoV‐2 infected patients. Panel A. Case n.8 demonstrated grossly intraparenchymal bilateral multifocal haemorrhages, one of which extended to the cerebral ventricles. Panel B. Gross examination of case n. 10 revealed multiple areas of white matter discoloration (arrows). Panel C. Histology of case n.5 demonstrated a thrombus in a leptomeningeal vein. This was the only thrombosis‐related finding in our series (H&E, 40x). Panel D and E. Microscopically, areas of white matter discoloration of case n. 10 contained microhaemorrhages (H&E, 200x) and acute inflammatory cells parenchymal infiltrates (H&E, 400x); Panel F and G. CD68‐KP1 immunostaining demonstrated microglial proliferation (F, anti‐CD68‐KP1, 200x), and immunohistochemistry for myeloperoxidase (G, anti‐MPO, 400x) highlighted the neutrophilic infiltrate in brain parenchyma. [file NAN-48-0-s001.jpg]
